# Self-reported symptoms of chronic cough and breathlessness in working-age men in the city of Izhevsk, Russia: associations with cardiovascular disease risk factors and comorbidities

**DOI:** 10.1136/bmjresp-2015-000104

**Published:** 2015-12-22

**Authors:** Sarah Cook, Jennifer K Quint, Maxim Vasiljev, David A Leon

**Affiliations:** 1 Department of Non Communicable Disease Epidemiology, London School of Hygiene & Tropical Medicine, London, UK; 2 Department of Respiratory Epidemiology, Occupational Medicine and Public Health, National Heart and Lung Institute, Imperial College London, London, UK; 3 Izhevsk State Medical Academy, Izhevsk, Russia; 4 Arctic University of Norway, UiT, Tromsø, Norway

**Keywords:** Clinical Epidemiology, COPD epidemiology

## Abstract

**Introduction:**

Very little is known about the prevalence of respiratory symptoms or their associations with other health conditions in Russia.

**Methods:**

Between 2008 and 2010, a sample of 983 men resident in Izhevsk, Russia, took part in a cross-sectional survey. Presence of respiratory symptoms was determined from self-report of chronic productive cough and breathlessness assessed using the British Medical Research Council (MRC) breathlessness scale. Self-reported physical and mental health were measured using the 12-Item Short-Form Health Survey (SF-12). Hypertension was assessed from mean blood pressure measured at the health check and/or self-reported use of antihypertensive medication. Other comorbidities were assessed from self-report. Logistic regression models were fitted assessing the association between respiratory symptoms and comorbidities. Linear regression models were fitted to investigate the association between respiratory symptoms and self-reported health scores. All models were adjusted for age, education and smoking status.

**Results:**

The age-standardised prevalence of cough and breathlessness was 20.9% (prevalence with breathlessness MRC grade 3 or above 3.7%). The majority of men with respiratory symptoms (87.3%) were current smokers. Cough and breathlessness were associated with substantially worse self-reported physical and mental health (test for trend with severity of breathlessness p<0.001). Those with chronic cough and grade 3 or above breathlessness had higher odds of having hypertension (OR 3.03; 95% CI 1.36 to 6.74), diabetes (OR 10.55; 95% CI 2.69 to 41.37), angina pectoris (OR 7.54; 95% CI 3.61 to 15.73), previous myocardial infarction (OR 7.61; 95% CI 2.10 to 27.4) and previous stroke (OR 6.61; 95% CI 1.75 to 23.34) compared with those without respiratory symptoms.

**Conclusions:**

The prevalence of respiratory symptoms was high. Strong associations were found between respiratory symptoms and cardiovascular comorbidities. These are of particular importance given the extremely high level of cardiovascular disease mortality in Russia.

**Key messages**

- The prevalence of respiratory symptoms in Izhevsk was high and associated with substantial cardiovascular comorbidity.
- The prevalence of smoking was extremely high even among those with severe breathlessness, suggesting the need for improved smoking cessation treatment in those with respiratory symptoms.
- This is one of the very few papers investigating the burden of respiratory disease in the Russian Federation and shows the need for further research into this neglected area.

## Introduction

Chronic respiratory diseases are a major cause of morbidity and mortality worldwide.^1^ ^2^

Tobacco smoking is an important risk factor for lung disease.^3^ Prevalence of smoking in Russia is very high among men, estimated at 60.2% in the 2009 Global Adult Tobacco Survey,^4^ and has shown no evidence of a decline over time,^5^ ^6^ thus suggesting that the burden of chronic respiratory disease in Russia is likely to be substantial. In addition, occupational exposure to vapours, dusts and fumes are likely to be important risk factors for respiratory diseases among industrial workers in Russia.^7^

Despite a high prevalence of smoking in Russia, very little attention has been paid to the burden of chronic respiratory problems in the Russian population. We are aware of only two studies investigating the prevalence of respiratory symptoms in the general population. Vietri *et al*^8^ found in the analyses of 5920 adults, aged 40 years or older in the 2011 Russia National Health and Wellness Survey, that 54% of respondents reported one or more symptoms of chronic respiratory disease (45% reported shortness of breath, 27% reported coughing up mucous, and 18% reported wheezing). Chuchalin *et al*^9^ found in a cross-sectional survey of 7164 adults from 12 regions in Russia that 25.7% reported an attack of wheezing or whistling with breathlessness and 8.6% reported symptoms of chronic bronchitis. Both previous studies suggest that the prevalence of chronic respiratory disease in Russia is high.

Russia has one of the highest levels of cardiovascular disease mortality in the world.^10^ There is evidence that respiratory disease and cardiovascular disease are linked, with associations found between cardiovascular morbidity and mortality and chronic obstructive pulmonary disease (COPD),^11–16^ asthma,^17–19^ and idiopathic pulmonary fibrosis.^20^ Cardiovascular disease is one of the main causes of death in both patients with COPD^11^ ^14^ ^16^ ^21^ and asthma.^22^ ^23^ The reasons for links between respiratory and cardiovascular diseases are not well understood. Some of these associations may be due to smoking as a common cause; however, systemic inflammation has also been suggested as a potential mechanism.^11^ ^16^ Despite the high prevalence of cardiovascular disease, to our knowledge, no previous studies have investigated the association between respiratory and cardiovascular morbidity in Russia.

The aim of this study was to investigate the prevalence of respiratory symptoms (chronic productive cough and breathlessness) and their associations with cardiovascular risk factors and self-reported comorbidities among working-age men in the city of Izhevsk, Russia.

## Methods

### Study sample

The study sample was 938 working-age men (25–60 years old) who were residents in the city of Izhevsk, an industrial city in Russia located 1300 km south east of Moscow, West of the Ural Mountains.

These men were participants in the Izhevsk Family Study 2 (IFS-2). This was a follow-up study of 1941 men living in Izhevsk originally recruited as population controls in a case–control study of the association between hazardous alcohol consumption and premature mortality in 2003–2006.^24^ The original controls were living men frequency matched by age to cases (men aged 25–54 years who had died in the past year) selected at random from the population list of Izhevsk in 2002. In IFS-2 (2008–2010), 1515 of these men were followed up, interviewed by a trained interviewer and invited to attend a health check. The health check conducted by a doctor included questions on medical history, three measurements of blood pressure and collection of a blood sample. There were four different doctors who conducted the health check. Participants were also given a self-completed questionnaire which included the 12-Item Short-Form Health Survey (SF-12) questions.^25^ For the majority of men, the health check took place in a polyclinic, but those who were unable to attend the clinic were offered the option of having this done in their own home.

**Figure 1:**
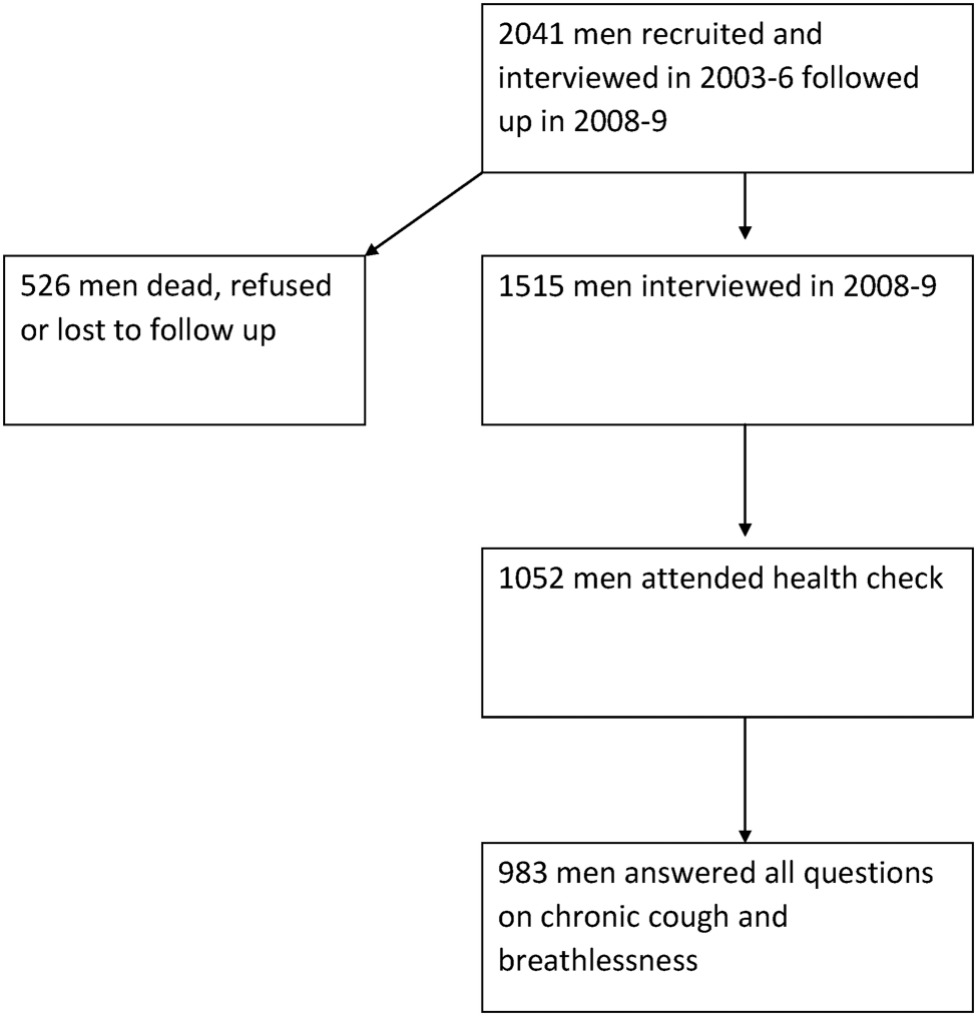
Flow chart of participants.

Out of the 1515/2041 men successfully reinterviewed as part of the IFS-2 study, 1052 attended the health check. For the purposes of this study, the sample was restricted to 983 men, with complete data collected on self-reported respiratory symptoms. A flow chart of participation in the study is shown in figure 1.

IFS-2 was approved by the Ethics Committees of the London School of Hygiene & Tropical Medicine and Izhevsk Medical Academy. Informed written consent was obtained from all participants to take part in the health check.

### Definition of respiratory symptoms

No spirometry data were collected at the health check; therefore, it was not possible to investigate prevalence of a specific disease such as COPD or asthma. However, data were collected on self-reported respiratory symptoms (chronic productive cough and breathlessness). Men who reported ‘Don't know/difficult to answer’ to any of the questions were coded as missing data on self-reported symptoms. The impact of this was assessed in sensitivity analyses.

Chronic productive cough was defined by one positive answer to the following questions: ‘Do you usually cough up any phlegm from your chest first thing in morning in winter?’, and/or ‘Do you usually cough up any phlegm from your chest, during the day or at night in winter?’, and/or ‘Do you bring up phlegm like this on most days for as much as 3 months each year?’.

Breathlessness was defined as a positive answer to one or more of the questions: (1) ‘Are you troubled by shortness of breath when hurrying on level ground or walking up a slight hill?’, and/or (2) ‘Do you get short of breath walking with people of your own age group on level ground?’, and/or (3) ‘Do you have to stop for breath when walking at your own pace on level ground?’, and/or (4) ‘Have you had attacks of wheezing or whistling in your chest at any time in the last 12 months?’, and/or (5) ‘Have you at any time in the past 12 months been woken at night by an attack of shortness of breath?’.

Breathlessness questions 1, 2 and 3 are part of the UK Medical Research Council (MRC) breathlessness scale, a validated tool for measuring disability associated with breathlessness,^26^ and were used to further divide men with respiratory symptoms into those with breathlessness grade 2 or less, and those with breathlessness grade 3 or higher. Question 4 was used to identify prevalence of wheeze and question 5, prevalence of nocturnal dyspnoea.

### Definition of exposures

#### Comorbidities

Comorbidities included were hypertension, diabetes, angina, previous myocardial infarction (MI) and previous stroke. All data on comorbidities were collected at the health check.

Three measurements of blood pressure were taken during the health check. Men were categorised as having hypertension if mean systolic blood pressure using the second and third blood pressure measurements was greater than 139 mm Hg or diastolic blood pressure was greater than 89 mm Hg and/or participants reported taking any antihypertensive medications either daily or sometimes.

Angina was determined from self-reported angina symptoms measured using the Rose angina questionnaire.^27^

Classification of diabetes mellitus, MI and stroke were all assessed from self-report of these conditions.

#### Self-reported health

Self-reported physical and mental health were measured using the SF-12, a standardised instrument for measuring self-reported health, derived as a shorter alternative to the SF-36.^25^ In accordance with standard practice, two summary scales were derived from these questions: a physical health component score and a mental health component score. Both of these summary scores ranged from 0 to 100.

#### Cardiovascular risk factors

Other exposures of interest were body mass index (BMI; kg/m^2^), total cholesterol, high-density lipoprotein cholesterol (HDL-C), and triglycerides measured in mmol/L, and B-type natriuretic peptide (BNP) measured in pg/mL. BNP is a biomarker of heart failure which is released with abnormal cardiac wall strain. Levels may also be raised for other reasons, including age, myocardial ischaemia, atrial fibrillation and renal dysfunction.^28^ ^29^ BNP is an independent predictor of cardiovascular morbidity and mortality,^30^ ^31^ and has been found to be strongly associated with hazardous alcohol consumption in this population.^32^ The distributions of triglycerides and BNP were skewed; therefore, these variables were transformed using the natural logarithm.

### Potential confounding variables

#### Age

Age was categorised into 5-year age groups and used as a continuous variable in analyses.

#### Education

Three categories of education were used: incomplete secondary, secondary, and higher or incomplete higher education. Owing to data sparsity, education was used as a continuous variable in analyses.

#### Smoking

Self-reported smoking status was categorised into five groups: never smoked, ex-smoker, current smoker 1–5 cigarettes per day, current smoker 11–20 cigarettes per day and current smoker >20 cigarettes per day.

#### Alcohol consumption

Alcohol consumption was considered as a potential confounder as it is strongly associated with smoking status and is also an important predictor of general health status and cardiovascular mortality in Russia.^24^ ^33^ ^34^

Two aspects of alcohol consumption in the past 12 months were considered: quantity of alcohol consumed and hazardous drinking pattern. Quantity of alcohol consumed was measured using total volume of ethanol consumed in the past year calculated from questions on the frequency and usual quantity of beer, wine and spirits consumed. Using an approach developed in our previous work, men were also classified as non-drinkers, non-hazardous drinkers and hazardous drinkers.^24^ ^35^ Men were classified as hazardous drinkers if they reported twice weekly or more frequency of hangover and/or excessive drunkenness and/or sleeping in clothes at night because of drunkenness and/or failing their family or personal obligations because of drinking and/or drinking non-beverage alcohols (sources of ethanol not intended for drinking such as eau de cologne) and/or one or more episodes of zapoi (a period of 2 or more days of drinking during which a participant is withdrawn from normal social life).

#### Medication use

Men were asked to report all medications they were taking at the time of the health check coded by a cardiologist. Use of medication for a respiratory problem was assessed by self-reported use of medications classified as bronchodilators, glucocorticoids and mucolytics. Data were not specifically collected on route of admission (ie, oral or inhaled).

### Statistical analysis

The prevalence of chronic cough and breathlessness was directly standardised by age using the 2013 European Standard Population aged 25–59 years.

Logistic regression models were fitted with each of the cardiovascular comorbidities as the outcome (hypertension, diabetes, angina, MI and stroke) and respiratory symptoms categorised by degree of breathlessness as the exposure variable.

It was necessary to limit, where possible, the number of confounders which could be included in the models because of sparsity of data for four of the outcomes (diabetes, angina, MI and stroke). Models were adjusted for age and education as a priori confounders and then additionally for smoking status. Smoking was considered as a potential confounder because it is strongly associated with both respiratory and cardiovascular diseases. However, smoking status could also be affected by both respiratory and cardiovascular diseases, and as people may stop smoking due to illness, adjustment could also lead to spurious associations due to reverse causality. Models were, therefore, considered with and without adjustment for smoking status. Although alcohol consumption was considered a priori as a potential confounder, no strong associations were found between the two different measures of alcohol use and respiratory symptoms in univariate analysis; therefore, alcohol consumption was not included as a confounder in the final model.

The associations between self-reported physical and mental health components of the SF-12 and serum lipids (total cholesterol, HDL-C and triglycerides), BNP and BMI were assessed using linear regression models with self-reported respiratory symptoms as exposure variables. Models were adjusted for age, education and smoking status.

### Sensitivity analysis

There were 63 men who answered “don't know” to at least one question on their respiratory symptoms. In the main analyses, these men were excluded as missing. However, a sensitivity analysis was carried out first recategorising ‘don't know’ answers to ‘no’ for that particular question, and then recategorising ‘don't know’ answers to ‘yes’ for that particular question. Analyses were rerun under these two alternative scenarios.

## Results

Overall data were available on symptoms of chronic cough and breathlessness for 983 men. There were no differences in these men with respect to age, education, smoking status or alcohol use from data available on respiratory symptoms compared with all the 1515 men who were interviewed in the IFS-2 study.

In the population, overall prevalence of chronic cough was 44.0% (n=432), breathlessness (grade 2 or above) was 33.9% (n=333), breathlessness (grade 3 or above) was 8.9% (n=87), wheeze was 4.3% (n=42), and nocturnal dyspnoea was 3.2% (n=31).There were 204 men (20.8%) who reported symptoms of both cough and breathlessness (age-standardised prevalence 20.9% (95% CI 18.4% to 23.5%)). Among men with chronic cough, 49 (24.0%) reported grade 3 or above breathlessness. The prevalence of wheeze among those with chronic cough and breathlessness was 16.7% (34 men) and nocturnal dyspnoea 11.4% (23 men). The prevalence of respiratory symptoms by smoking status is shown in table 1.

**Table 1:** Prevalence of self-reported respiratory symptoms by smoking status

Respiratory symptom	Non-smoker		Ex-smoker		Current smoker		All men	
	N	(%)	N	(%)	N	(%)	N	(%)
Chronic productive cough	24	(12.6)	40	(21.7)	368	(60.6)	432	(44.0)
MRC breathlessness grade 2	34	(17.8)	39	(21.2)	173	(28.5)	246	(25.1)
MRC breathlessness grade 3 and above	11	(5.8)	18	(9.8)	58	(9.6)	87	(8.9)
Wheeze	5	(2.6)	4	(2.2)	33	(5.4)	42	(4.3)
Nocturnal dyspnoea	6	(3.1)	6	(3.3)	19	(3.1)	31	(3.2)
Chronic cough and breathlessness*	9	(4.7)	17	(9.2)	178	(29.3)	204	(20.8)
Total	191	(100)	184	(100)	607	(100)	982†	(100)

*Breathlessness defined as MRC breathless grade 2 or above and/or wheeze and/or nocturnal dyspnoea.

†Smoking status missing for one man.

MRC, Medical Research Council.

The level of use of medications for respiratory problems was very low. Ten men reported using bronchodilators (seven with symptoms of chronic cough and breathlessness). Four men reported using glucocorticoids (two with symptoms of chronic cough and breathlessness) and seven men reported use of mucolytics (three with symptoms of chronic cough and breathlessness).

The majority of men with chronic cough and breathlessness were current smokers (87.3%).There was no evidence of a systematic difference in smoking behaviour by grade of breathlessness (p=0.54) despite a higher percentage of ex-smokers in those with breathlessness grade 3 or above compared with those with grade 2 breathlessness (6.5% vs 14.3%). The distribution of men with and without symptoms of chronic cough and breathlessness by sociodemographic factors, smoking, cardiovascular risk factors, self-reported health and comorbidities is shown in table 2. There was strong evidence of an association between self-reported symptoms of chronic cough and breathlessness and education. The proportion of men with higher education decreased as severity of breathlessness increased. Different patterns emerged for the two measures of alcohol use: total volume consumed was highest among men with chronic cough and MRC breathlessness of grade 2 or less, but lowest among men with chronic cough and MRC breathlessness grade 3 or less. Conversely, the proportion of hazardous drinkers in the sample increased with severity of breathlessness. However, there was only weak evidence for an association with either measure of alcohol use and self-reported symptoms of chronic cough and breathlessness.

**Table 2:** Distribution of sample by chronic cough and MRC breathlessness grade, sociodemographic variables, smoking, cardiovascular risk factors and health status

	No respiratory symptoms		Chronic cough and breathlessness			
			≤Grade 2 breathlessness		≥Grade 3 breathlessness	
	N, mean or median	(%,SD or IQR)	N, mean or median	(%, SD or IQR)	N, mean or median	(%, SD or IQR)
Age (years)						
25–29	10	(1.3)	3	(1.9)	0	(0.0)
30–34	58	(7.5)	12	(7.7)	2	(4.1)
35–39	63	(8.1)	20	(12.9)	4	(8.2)
40–44	96	(12.3)	14	(9.0)	3	(6.1)
45–49	160	(20.5)	29	(18.7)	3	(6.1)
50–54	187	(24.0)	34	(21.9)	12	(24.5)
55–60	205	(26.3)	43	(27.7)	25	(51.0)
χ^2^ (df)	23.13 (12) p=0.03					
Education						
Incomplete secondary	31	(4.0)	9	(5.8)	3	(6.1)
Secondary	556	(71.4)	123	(79.4)	43	(87.8)
Higher or incomplete higher	192	(24.7)	23	(14.8)	3	(6.1)
χ^2^ (df)	15.5 (4) p=0.004					
Smoking status (missing=1)						
Never smoked	182	(23.4)	7	(4.5)	2	(4.1)
Ex-smoker	167	(21.5)	10	(6.5)	7	(14.3)
Current smoker (1–10/day)	81	(10.4)	25	(16.1)	7	(14.3)
Current smoker (11–20/day)	274	(35.2)	82	(52.9)	25	(51.0)
Current smoker (>20/day)	74	(9.5)	31	(20.0)	8	(16.3)
χ^2^ (df)	75.9 (8) p<0.001					
Total volume of ethanol (litres per year) (missing=13)						
Median (IQR)	4.0	(1.2–10.1)	5.9	(1.7–12.5)	3.1	(1.2–17.3)
Kruskal-Wallis	p=0.13					
Hazardous drinker (missing=12)						
Non-drinker	104	(13.5)	18	(11.7)	6	(12.2)
Drinker (non-hazardous)	602	(78.4)	117	(76.0)	34	(69.4)
Drinker (hazardous)	62	(8.1)	19	(12.3)	9	(18.4)
χ^2^ (df)	8.03 (4) p=0.09					
Body mass index (missing=4)						
Mean (SD)	26.2	(4.2)	26.4	(4.7)	27.8	(5.2)
Test for trend	p=0.04					
Total cholesterol mmol/L (missing=66)						
Mean (SD)	5.44	(1.02)	5.27	(0.96)	5.22	(1.01)
Test for trend	p=0.03					
HDL-C mmol/L (missing=66)						
Mean (SD)	1.44	(0.44)	1.40	(0.48)	1.36	(0.40)
Test for trend	p=0.14					
Log triglycerides (missing=82)						
Mean (SD)	0.29	(0.51)	0.26	(0.50)	0.30	(0.52)
Test for trend	p=0.73					
Log BNP pg/mL (missing=74)						
Mean (SD)	2.42	(0.87)	2.59	(1.03)	2.99	(0.96)
Test for trend	p<0.001					
SF-12 (missing=15)						
Mean Physical health score (SD)	48.8	(7.7)	45.2	(7.7)	36.1	(9.3)
Test for trend	p<0.001					
Mean Mental health score (SD)	49.2	(8.6)	47.9	(8.8)	43.6	(10.3)
Test for trend	p<0.001					
Hypertension (missing=2)						
Yes	477	(61.4)	97	(62.6)	41	(83.7)
χ^2^ (df)	9.79(2) p=0.007					
Diabetes (Missing=7)						
Yes	9	(1.2)	3	(2.0)	4	(8.3)
χ^2^ (df)	14.54 (2) p=0.001					
Angina (missing=9)						
Yes	43	(5.6)	24	(15.6)	14	(29.2)
χ^2^ (df)	45.7 (2) p<0.001					
Myocardial infarction (missing=2)						
Yes	11	(1.4)	2	(1.3)	5	(10.2)
χ^2^ (df)	20.1 (2) p<0.001					
Stroke (missing=3)						
Yes	8	(1.0)	1	(0.70)	4	(8.2)
χ^2^ (df)	18.6 (2) p<0.001					
Total	779	(100)	155	(100)	49	(100)

BNP, B-type natriuretic peptide; HDL-C, high-density lipoprotein cholesterol; SF-12, 12-Item Short-Form Health Survey.

The cross-sectional associations between self-reported chronic cough and breathlessness and comorbidities are shown in table 3. The odds of hypertension, diabetes, angina, MI and stroke were all raised in men with self-reported cough with breathlessness of grade 3 or above compared with men with no respiratory symptoms. There was strong evidence that the odds of diabetes, angina and MI, and good evidence that the odds of hypertension and stroke increased with severity of breathlessness. These associations remained on adjusting for smoking.

**Table 3:** Association between self-reported chronic cough and breathlessness symptoms and other comorbidities

		No respiratory symptoms	Chronic cough and breathlessness		
Self-reported comorbidity		Odds ratio (95% CI)	≤Grade 2 breathlessness	≥Grade 3 breathlessness	Test for trend
Hypertension (n=980)	Model 1	1 (ref)	1.12 (0.77 to 1.62)	2.84 (1.29 to 6.26)	p=0.04
	Model 2	1 (ref)	1.19 (0.81 to 1.74)	3.03 (1.36 to 6.74)	p=0.02
Diabetes (n=975)	Model 1	1 (ref)	1.86 (0.49 to 7.03)	7.27 (2.05 to 25.85)	p=0.008
	Model 2	1 (ref)	2.91 (0.68 to 12.37)	10.55 (2.69 to 41.37)	p=0.002
Angina (n=973)	Model 1	1 (ref)	3.29 (1.91 to 5.65)	6.74 (3.31 to 13.75)	p<0.001
	Model 2	1 (ref)	3.73 (2.09 to 6.66)	7.54 (3.61 to 15.73)	p<0.001
Myocardial infarction (n=980)*	Model 1	1 (ref)	0.91 (0.20 to 4.20)	5.18 (1.65 to 16.22)	p=0.04
	Model 2	1 (ref)	1.40 (0.27 to 7.17)	7.61 (2.10 to 27.49)	p=0.008
Stroke (n=979)*	Model 1	1 (ref)	0.71 (0.09 to 5.77)	7.76 (2.09 to 28.88)	p=0.03
	Model 2	1 (ref)	0.60 (0.07 to 5.01)	6.61 (1.73 to 25.34)	p=0.06

Model 1: adjusted for age and education.

Model 2: model 1 + smoking status.

*For myocardial infarction and stroke, the smoking variable was recoded so that the category >20 cigarettes/day merged with 11–20 cigarettes per day; otherwise 113 observations dropped due to perfect prediction.

The cross-sectional associations between self-reported chronic cough and breathlessness symptoms, self-reported health, serum lipids, BNP, and BMI are shown in table 4. There was strong evidence for a substantial decrease in physical self-reported health score with severity of breathlessness symptoms as men with cough and grade 3 breathlessness had a mean physical health score 11.58 points (95% CI 9.34 to 13.83) lower than men who did not report any respiratory symptoms. There was also a corresponding but smaller inverse trend in self-reported mental health (men with grade 3 breathlessness reported a mean mental health score 5.90 points lower than men with no respiratory symptoms (95% CI 8.51 to 3.29)). There was some evidence for a negative association with total cholesterol (test for trend p=0.06) and HDL-C (test for trend p=0.01) and respiratory symptoms with evidence of a decrease with increasing severity of breathlessness. Mean BMI was higher in men with chronic cough and breathlessness; there was strong evidence for a positive trend with severity of breathlessness (test for trend p<0.001). There was also good evidence of a positive trend in log BNP with severity of breathlessness (test for trend p=0.006). There was no evidence for an association between respiratory symptoms and triglycerides (test for trend p=0.56).

**Table 4:** Association between self-reported chronic cough and breathlessness symptoms and self-reported health, lipids, BNP and body mass index

		No respiratory symptoms	Chronic cough and breathlessness		Test for trend
			≤Grade 2 breathlessness	≥Grade 3 breathlessness	
		Coefficient (95% CI)			
SF-12 PCS (n=967)	Model 1	1 (ref)	−3.49 (−4.80 to −2.18)	−11.56 (−13.78 to −9.33)	p<0.001
	Model 2	1 (ref)	−3.60 (−4.95 to −2.25)	−11.58 (−13.83 to −9.34)	p<0.001
SF-12 MCS (n=967)	Model 1	1 (ref)	−1.36 (−2.88 to 0.16)	−5.97 (−8.55 to −3.39)	p<0.001
	Model 2	1 (ref)	−1.33 (−2.90 to 0.24)	−5.90 (−8.51 to −3.29)	p<0.001
Total cholesterol (n=916)	Model 1	1 (ref)	−0.16 (−0.33 to 0.02)	−0.25 (−0.55 to 0.05)	p=0.02
	Model 2	1 (ref)	−0.13 (−0.31 to 0.06)	−0.23 (−0.53 to 0.08)	p=0.06
HDL-C (n=916)	Model 1	1 (ref)	−0.05 (−0.13 to 0.03)	−0.10 (−0.23 to 0.03)	p=0.07
	Model 2	1 (ref)	−0.08 (−0.16 to 0.002)	−0.13 (−0.26 to 0.01)	p=0.01
Log triglycerides (n=900)	Model 1	1 (ref)	−0.02 (−0.11 to 0.07)	0.02 (−0.14 to 0.17)	p=0.88
	Model 2	1 (ref)	0.01 (−0.08 to 0.11)	0.04 (−0.11 to 0.20)	p=0.56
Log BNP (n=908)	Model 1	1 (ref)	0.16 (0.003 to 0.31)	4.14 (0.16 to 0.67)	p=0.001
	Model 2	1 (ref)	0.11 (−0.05 to 0.26)	0.37 (0.12 to 0.63)	p=0.006
Body mass index (n=978)	Model 1	1 (ref)	0.37 (−0.38 to 1.12)	1.86 (0.60 to 3.12)	p=0.01
	Model 2	1 (ref)	0.95 (0.19 to 1.70)	2.29 (1.05 to 3.53)	p<0.001

Model 1: adjusted for age and education.

Model 2: model 1 + smoking status.

BNP, B-type natriuretic peptide; HDL-C, high-density lipoprotein cholesterol; MCS, mental health component score; PCS, physical health component score; SF-12, 12-Item Short-Form Health Survey.

### Sensitivity analyses

When men who answered ‘don't know’ to any particular question were included, the sample size was 1046. Assuming that all men who answered ‘don't know’ did not have the symptom, the prevalence of cough was 45.4%, any breathlessness was 34.5%, breathlessness grade 3 or above was 9.3%, and for both cough and breathlessness was 21.9%. Under the alternative scenario that all men who answered ‘don't know’ did have that symptom, the prevalence of cough was 45.8%, any breathlessness was 37.2%, breathlessness grade 3 or above was 11.6%, and for both cough and breathlessness was 23.5%. The substantive associations between self-reported cough and breathlessness and the other health outcomes were not altered under either scenario with the exception that in both the sensitivity analyses, no associations were seen between self-reported respiratory symptoms and total cholesterol (data not shown).

## Discussion

In this sample of working-age men living in the industrial city of Izhevsk, the age-standardised prevalence of self-reported symptoms of chronic cough and breathlessness was 20.9% (age-standardised prevalence of chronic cough with grade 3 or above breathlessness was 3.7%). The prevalence of chronic cough was 44.0% and breathlessness grade 2 or above was 33.9%. This is high compared with a prevalence of self-reported cough and/or breathlessness from the BREATHE study of 12.2% among 31 418 men living in 11 countries in the Middle East and North Africa.^36^ The prevalence of cough and breathlessness among men in Izhvesk was higher than for any of the countries included in the BREATHE study (range 6.1% in the United Arab Emirates to15.9% in Pakistan). Chronic cough and breathlessness are both symptoms of COPD and the high prevalence of these symptoms suggests prevalence of COPD is likely to be high in this population. This is consistent with the one previous study which has investigated prevalence of COPD in Russia using spirometry; the study estimated overall prevalence in both men and women of 15.3%^9^ compared with an international prevalence of 10.1% in the BOLD study (11.8% in men).^37^ Further studies in Russia using spirometry are needed to provide more reliable estimates of the burden of COPD.

In this study, self-reported symptoms of cough and breathlessness were associated with poorer self-reported physical and mental health as assessed by the SF-12. There was a particularly strong association with physical health; men with chronic cough and grade 3 breathlessness or above had a physical health score 11.6 points (95% CI 9.3 to 13.8) lower than men without any respiratory symptoms, indicating the severe impact of impaired respiratory function on quality of life for these men. Men with chronic cough and grade 3 or above breathlessness also had much higher odds of several comorbidities (hypertension, diabetes, angina pectoris, MI and stroke) compared with men without respiratory symptoms, even after adjusting for smoking status. These strong associations are consistent with the findings from other countries that respiratory diseases, in particular COPD, are associated with substantial cardiovascular morbidity.^11–13^ ^15^ ^17–20^ The association between respiratory symptoms and cardiovascular morbidity has not been investigated previously in Russia. The strong associations with other health conditions found in this study have implications for management of patients with respiratory symptoms; these patients could benefit from identification and treatment of comorbidities. Along with increased odds of cardiovascular comorbidities, men in Izhvesk with respiratory symptoms also had on average a worse cardiovascular risk profile with higher BMI and lower HDL-C levels; however, total cholesterol was also lower and no association was found with triglycerides. There was also strong evidence of higher levels of BNP with respiratory symptoms. Some of this association may be due to misclassification of heart failure as a respiratory problem in this study since breathlessness is a symptom of both; however, this finding is consistent with previous studies which have found both higher levels of BNP among patients with COPD compared with healthy controls^38^ and that heart failure is a common comorbidity among those with COPD.^39^ There were only weak associations found in this study between alcohol consumption and respiratory symptoms despite strong associations between hazardous alcohol consumption and blood pressure, cardiovascular disease and BNP.^32^ ^33^ ^40–43^ Given the very high levels of cardiovascular mortality in Russia, the identification of patients with respiratory problems as a potentially high-risk group who may benefit from primary and secondary prevention could be particularly important. Conversely, respiratory function should also be investigated among those with cardiovascular disease.

In this study the majority of men (87.3%) who reported respiratory symptoms continued to smoke. This was the case even for men experiencing more severe breathlessness (81.6% current smokers). Smoking cessation is a key part of treatment for many respiratory conditions. This is the single most effective treatment for COPD with an important role in slowing the disease progression.^14^ ^44^ An estimated 62% of patients with COPD in the UK are current smokers estimated from a random sample of patients with COPD in the UK population registered with general practitioner practices in the Clinical Practice Research Datalink.^45^ Although not directly comparable, the findings from Izhvesk are consistent with findings from this study and studies in clinical populations that a large proportion of those with COPD continue to smoke despite their disease.^46^ Smoking cessation treatment has been found to be effective for reducing smoking in patients with COPD^46–49^ and should be offered routinely to those reporting respiratory symptoms.

There were several limitations to this study. First spirometry was not carried out at the health check. Self-reported symptoms of cough and breathlessness were used here to make some inference about chronic respiratory disease burden overall and its associations with cardiovascular comorbidities in this population. We would like to use these findings to make some inferences particularly about COPD, but this should be done with caution given that the case definition used here is less specific than COPD as determined by spirometry, and that some men with cough and breathlessness in this study may have had other lung conditions such as asthma or tuberculosis. In addition, all comorbidities were also self-reported which is an additional source of measurement error. A further limitation in considering the prevalence estimates is that the study population was a small subset of men aged 25–59 years living in the city of Izhevsk; therefore, the results on prevalence are not generalisable to women, older men or to men living elsewhere in Russia and estimates of prevalence are likely to be imprecise. However, the finding that prevalence of chronic cough and breathlessness is high is consistent with findings from other studies in Russia.^8^ ^9^ There may also have been some selection bias in the study since only 65% of men interviewed at IFS-2 went on to attend the health check. However, no differences were found between men included in this study and all men taking part in IFS-2.

A further limitation was that no data were collected about clinical diagnoses and data collected on treatment were limited. Men were asked about what medications they were taking. However, this question did not refer specifically to inhalers and it is possible many men did not report these. Only 10 men reported the use of bronchodilators in the sample overall, but it is not possible to disentangle whether this is due to very low levels of treatment with bronchodilators or under-reporting of use of these medications. There seems to be a very low level of use of bronchodilators given that some men are likely to have been prescribed bronchodilators for asthma, though it is worth noting that bronchodilators could be expensive for a substantial proportion of the population as Russian patients need to pay the full cost of their medications. It was, however, possible to look at the prevalence of smoking cessation, an important aspect of treatment for respiratory disease. The high proportion of current smokers with respiratory symptoms supports findings from medication data that treatment levels in this population are low. Finally there was a low prevalence of several of the comorbidities included, in particular stroke, diabetes and MI; therefore, results are based on small numbers and a limited number of potential confounders were included. Despite these limitations, the finding of a high level of self-reported chronic cough and breathlessness in this study is important given the very limited data available on the burden of chronic respiratory conditions in the Russian Federation.

In conclusion, in this sample of working-age men living in Izhevsk, the prevalence of chronic cough and breathlessness was high and associated with worse self-reported physical and mental health and higher levels of cardiovascular comorbidities. Despite the importance of smoking cessation in treatment for respiratory diseases, the majority of men with chronic cough and breathlessness in this population continued to smoke, thus showing that this is not being tackled sufficiently. Chronic respiratory disease is an important public health issue for Russia, but the low levels of research published suggest this is a neglected area. More research is needed, specifically around the levels of diagnosis and treatment in Russia. The association between respiratory symptoms and cardiovascular comorbidity is of particular importance given the extremely high levels of cardiovascular mortality in Russia.

## References

[1] Lozano R, Naghavi M, Foreman K Global and regional mortality from 235 causes of death for 20 age groups in 1990 and 2010: a systematic analysis for the Global Burden of Disease Study 2010. Lancet 2012;380:2095–128. doi:10.1016/S0140-6736(12)61728-0 23245604 10.1016/S0140-6736(12)61728-0 PMC10790329

[2] Vos T, Flaxman AD, Naghavi M Years lived with disability (YLDs) for 1160 sequelae of 289 diseases and injuries 1990–2010: a systematic analysis for the Global Burden of Disease Study 2010. Lancet 2012;380:2163–96. doi:10.1016/S0140-6736(12)61729-2 23245607 10.1016/S0140-6736(12)61729-2 PMC6350784

[3] Centers for Disease Control and Prevention (US), National Center for Chronic Disease Prevention and Health Promotion (US), Office on Smoking and Health (US). How tobacco smoke causes disease: the biology and behavioral basis for smoking-attributable disease: a report of the Surgeon General. Atlanta: Centers for Disease Control and Prevention (US), 2010. 21452462

[4] Giovino GA, Mirza SA, Samet JM Tobacco use in 3 billion individuals from 16 countries: an analysis of nationally representative cross-sectional household surveys. Lancet 2012;380:668–79. doi:10.1016/S0140-6736(12)61085-X 22901888 10.1016/S0140-6736(12)61085-X

[5] Perlman F, Bobak M, Gilmore A Trends in the prevalence of smoking in Russia during the transition to a market economy. Tob Control 2007;16:299–305. doi:10.1136/tc.2006.019455 17897987 10.1136/tc.2006.019455 PMC2598552

[6] Roberts B, Gilmore A, Stickley A Changes in smoking prevalence in 8 countries of the former Soviet Union between 2001 and 2010. Am J Public Health 2012;102:1320–8. doi:10.2105/AJPH.2011.300547 22594739 10.2105/AJPH.2011.300547 PMC3478012

[7] Mazitova NN, Saveliev AA, Berheeva ZM COPD and occupation: a retrospective cohort study of industrial workers. Arh Hig Rada Toksikol 2012;63:345–56. doi:10.2478/10004-1254-63-2012-2178 23152384 10.2478/10004-1254-63-2012-2178

[8] Vietri J, Ertl S, Isherwood G Symptoms of COPD in urban Russia among adults 40 years and older. Value Health 2013;16:A375 doi:10.1016/j.jval.2013.08.303

[9] Chuchalin AG, Khaltaev N, Antonov NS Chronic respiratory diseases and risk factors in 12 regions of the Russian Federation. Int J Chron Obstruct Pulmon Dis 2014;9:963–74. doi:10.2147/COPD.S67283 25246783 10.2147/COPD.S67283 PMC4166963

[10] World Health Organisation. Cardiovascular diseases mortality: age-standardized death rate per 100 000 population, 2000–2012. http://gamapserver.who.int/gho/interactive_charts/ncd/mortality/cvd/atlas.htmlDate of access 14/12/15.

[11] Sin DD, Man SF Chronic obstructive pulmonary disease: a novel risk factor for cardiovascular disease. Can J Physiol Pharmacol 2005;83:8–13. doi:10.1139/y04-116 15759045 10.1139/y04-116

[12] Finkelstein J, Cha E, Scharf SM Chronic obstructive pulmonary disease as an independent risk factor for cardiovascular morbidity. Int J Chron Obstruct Pulmon Dis 2009;4:337–49. doi:10.2147/COPD.S6400 19802349 10.2147/copd.s6400 PMC2754086

[13] Divo M, Cote C, de Torres JP Comorbidities and risk of mortality in patients with chronic obstructive pulmonary disease. Am J Respir Crit Care Med 2012;186:155–61. doi:10.1164/rccm.201201-0034OC 22561964 10.1164/rccm.201201-0034OC

[14] Berry CE, Wise RA Mortality in COPD: causes, risk factors, and prevention. COPD 2010;7:375–82. doi:10.3109/15412555.2010.510160 20854053 10.3109/15412555.2010.510160 PMC7273182

[15] Mannino DM, Thorn D, Swensen A Prevalence and outcomes of diabetes, hypertension and cardiovascular disease in COPD. Eur Respir J 2008;32:962–9. doi:10.1183/09031936.00012408 18579551 10.1183/09031936.00012408

[16] Fabbri LM, Luppi F, Beghe B Complex chronic comorbidities of COPD. Eur Respir J 2008;31:204–12. doi:10.1183/09031936.00114307 18166598 10.1183/09031936.00114307

[17] Iribarren C, Tolstykh IV, Eisner MD Are patients with asthma at increased risk of coronary heart disease? Int J Epidemiol 2004;33:743–8. doi:10.1093/ije/dyh081 15131088 10.1093/ije/dyh081

[18] Iribarren C, Tolstykh IV, Miller MK Adult asthma and risk of coronary heart disease, cerebrovascular disease, and heart failure: a prospective study of 2 matched cohorts. Am J Epidemiol 2012;176:1014–24. doi:10.1093/aje/kws181 23139248 10.1093/aje/kws181

[19] Tattersall MC, Guo M, Korcarz CE Asthma predicts cardiovascular disease events: the multi-ethnic study of atherosclerosis. Arterioscler Thromb Vasc Biol 2015;35:1520–5. doi:10.1161/ATVBAHA.115.305452 25908767 10.1161/ATVBAHA.115.305452 PMC4441553

[20] Dalleywater W, Powell HA, Hubbard RB Risk factors for cardiovascular disease in people with idiopathic pulmonary fibrosis: a population-based study. Chest 2015;147:150–6. doi:10.1378/chest.14-0041 25121965 10.1378/chest.14-0041

[21] Hansell AL, Walk JA, Soriano JB What do chronic obstructive pulmonary disease patients die from? A multiple cause coding analysis. Eur Respir J 2003;22:809–14. doi:10.1183/09031936.03.00031403 14621089 10.1183/09031936.03.00031403

[22] Soto-Campos JG, Plaza V, Soriano JB “Causes of death in asthma, COPD and non-respiratory hospitalized patients: a multicentric study.” BMC Pulm Med 2013;13:73 doi:10.1186/1471-2466-13-73 24321217 10.1186/1471-2466-13-73 PMC4029295

[23] Bellia V, Pedone C, Catalano F Asthma in the elderly: mortality rate and associated risk factors for mortality. Chest 2007;132:1175–82. doi:10.1378/chest.06-2824 17890479 10.1378/chest.06-2824

[24] Leon DA, Saburova L, Tomkins S Hazardous alcohol drinking and premature mortality in Russia: a population based case-control study. Lancet 2007;369:2001–9. doi:10.1016/S0140-6736(07)60941-6 17574092 10.1016/S0140-6736(07)60941-6

[25] Ware J Jr, Kosinski M, Keller SD A 12-Item Short-Form Health Survey: construction of scales and preliminary tests of reliability and validity. Med Care 1996;34:220–33. doi:10.1097/00005650-199603000-00003 8628042 10.1097/00005650-199603000-00003

[26] Stenton C The MRC breathlessness scale. Occup Med (Lond) 2008;58:226–7. doi:10.1093/occmed/kqm162 18441368 10.1093/occmed/kqm162

[27] Rose G, McCartney P, Reid DD Self administration of a questionnaire on chest pain and intermittent claudication. Br J Prev Soc Med 1977;31:42–8. 856370 10.1136/jech.31.1.42 PMC478990

[28] Cowie MR, Jourdain P, Maisel A Clinical applications of B-type natriuretic peptide (BNP) testing. Eur Heart J 2003;24:1710–18. doi:10.1016/S0195-668X(03)00476-7 14522565 10.1016/s0195-668x(03)00476-7

[29] Noman A, George J, Struthers A A new use for B-type natriuretic peptide: to detect myocardial ischaemia in non-heart failure patients. Br J Diabetes Vasc Dis 2010;10:78–82. doi:10.1177/1474651409344927

[30] Linssen GC, Bakker SJ, Voors AA N-terminal pro-B-type natriuretic peptide is an independent predictor of cardiovascular morbidity and mortality in the general population. Eur Heart J 2010;31:120–7. doi:10.1093/eurheartj/ehp420 19854731 10.1093/eurheartj/ehp420

[31] Wang TJ, Larson MG, Levy D Plasma natriuretic peptide levels and the risk of cardiovascular events and death. N Engl J Med 2004;350:655–63. doi:10.1056/NEJMoa031994 14960742 10.1056/NEJMoa031994

[32] Leon DA, Shkolnikov VM, Borinskaya S Hazardous alcohol consumption is associated with increased levels of B-type natriuretic peptide: evidence from two population-based studies. Eur J Epidemiol 2013;28:393–404. doi:10.1007/s10654-013-9808-9 23645505 10.1007/s10654-013-9808-9 PMC3672507

[33] Leon DA, Shkolnikov V, McKee M Alcohol increases circulatory disease in Russia: acute and chronic effects or misattribution of cause? Int J Epidemiol 2010;39: 1279–90. doi:10.1093/ije/dyq102 20591986 10.1093/ije/dyq102 PMC2972439

[34] Zaridze D, Brennan P, Boreham J Alcohol and cause-specific mortality in Russia: a retrospective case-control study of 48,557 adult deaths. Lancet 2009;373:2201–14. doi:10.1016/S0140-6736(09)61034-5 19560602 10.1016/S0140-6736(09)61034-5 PMC2715218

[35] Tomkins S, Saburova L, Kiryanov N Prevalence and socio-economic distribution of hazardous patterns of alcohol drinking: study of alcohol consumption in men aged 25–54 years in Izhevsk, Russia. Addiction 2007;102:544–53. doi:10.1111/j.1360-0443.2006.01693.x 17362291 10.1111/j.1360-0443.2006.01693.x PMC1890567

[36] Tageldin MA, Nafti S, Khan JA Distribution of COPD-related symptoms in the Middle East and North Africa: results of the BREATHE study. Respir Med 2012;106 (Suppl 2):S25–32. doi:10.1016/S0954-6111(12)70012-4 23290701 10.1016/S0954-6111(12)70012-4

[37] Buist AS, McBurnie MA, Vollmer WM International variation in the prevalence of COPD (The BOLD Study): a population-based prevalence study. Lancet 2007;370:741–50. doi:10.1016/S0140-6736(07)61377-4 17765523 10.1016/S0140-6736(07)61377-4

[38] Bozkanat E, Tozkoparan E, Baysan O The significance of elevated brain natriuretic peptide levels in chronic obstructive pulmonary disease. J Int Med Res 2005;33: 537–44. doi:10.1177/147323000503300509 16222887 10.1177/147323000503300509

[39] Rutten FH, Cramer MJ, Lammers JW Heart failure and chronic obstructive pulmonary disease: an ignored combination?. Eur J Heart Fail 2006;8:706–11. doi:10.1016/j.ejheart.2006.01.010 16531114 10.1016/j.ejheart.2006.01.010

[40] Foerster M, Marques-Vidal P, Gmel G Alcohol drinking and cardiovascular risk in a population with high mean alcohol consumption. Am J Cardiol 2009;103:361–8. doi:10.1016/j.amjcard.2008.09.089 19166690 10.1016/j.amjcard.2008.09.089

[41] Klatsky AL Alcohol and cardiovascular health. Physiol Behav 2010;100:76–81. doi:10.1016/j.physbeh.2009.12.019 20045009 10.1016/j.physbeh.2009.12.019

[42] Marmot MG, Elliott P, Shipley MJ Alcohol and blood pressure: the INTERSALT study. BMJ 1994;308:1263–7. doi:10.1136/bmj.308.6939.1263 7802765 10.1136/bmj.308.6939.1263 PMC2540174

[43] Roerecke M, Rehm J Irregular heavy drinking occasions and risk of ischemic heart disease: a systematic review and meta-analysis. Am J Epidemiol 2010;171:633–44. doi:10.1093/aje/kwp451 20142394 10.1093/aje/kwp451

[44] Scanlon PD, Connett JE, Waller LA Smoking cessation and lung function in mild-to-moderate chronic obstructive pulmonary disease. The lung health study. Am J Respir Crit Care Med 2000;161 (2 Pt 1):381–90. doi:10.1164/ajrccm.161.2.9901044 10673175 10.1164/ajrccm.161.2.9901044

[45] Quint JK, Mullerova H, DiSantostefano RL Validation of chronic obstructive pulmonary disease recording in the Clinical Practice Research Datalink (CPRD-GOLD). BMJ Open 2014;4:e005540 doi:10.1136/bmjopen-2014-005540 10.1136/bmjopen-2014-005540 PMC4120321 25056980

[46] Tonnesen P Smoking cessation and COPD. Eur Respir Rev 2013;22:37–43. doi:10.1183/09059180.00007212 23457163 10.1183/09059180.00007212 PMC9487432

[47] Anthonisen NR, Connett JE, Kiley JP Effects of smoking intervention and the use of an inhaled anticholinergic bronchodilator on the rate of decline of FEV1: the lung health study. JAMA 1994;272:1497–505. doi:10.1001/jama.1994.03520190043033 7966841

[48] Murray RP, Connett JE, Rand CS Persistence of the effect of the Lung Health Study (LHS) smoking intervention over eleven years. Prev Med 2002;35:314–19. doi:10.1006/pmed.2002.1087 12453707 10.1006/pmed.2002.1087

[49] Strassmann R, Bausch B, Spaar A Smoking cessation interventions in COPD: a network meta-analysis of randomised trials. Eur Respir J 2009;34:634–40. doi:10.1183/09031936.00167708 19357145 10.1183/09031936.00167708

